# SYMPTOM BURDEN AND QUALITY OF LIFE FOR NURSING HOME RESIDENTS WITH DEMENTIA: UPLIFT TRIAL EARLY DATA

**DOI:** 10.1093/geroni/igac059.194

**Published:** 2022-12-20

**Authors:** John Cagle, Jessica Orth, Todd Becker, Peiyuan Zhang, Mary Ersek, Wanzhu Tu, Alex Floyd, Kathleen Unroe

**Affiliations:** University of Maryland, Baltimore, Baltimore, Maryland, United States; University of Maryland, Baltimore, Baltimore, Maryland, United States; University of Maryland, Baltimore, Baltimore, Maryland, United States; University of Maryland Baltimore, Baltimore, Maryland, United States; University of Pennsylvania, Philadelphia, Pennsylvania, United States; Indiana University School of Medicine, Indianapolis, Indiana, United States; Indiana University, Indianapolis, Indiana, United States; Indiana University Center for Aging Research, Regenstrief Institute, Inc., Indianapolis, Indiana, United States

## Abstract

Communication difficulties in nursing home (NH) residents with dementia make valid assessment of symptoms and quality-of-life (QOL) challenging. Thus, researchers and clinicians frequently rely on proxy-based measures. The End-of-life Dementia-Comfort Assessment in Dying (EOLD-CAD) and two single-item QOL measures (7-point item; 5-point item) have been used in several studies, though evaluation of their psychometric properties is limited. We used baseline data from an ongoing multi-site randomized trial (UPLIFT) to describe symptoms and QOL and examine the measures’ validity and reliability in 138 residents with moderate to severe dementia living at 16 facilities. Descriptive data and assessments of convergent validity and inter-rater reliability are provided. Based on assessments by 134 staff and 45 family, physical symptoms, physical distress, and emotional distress were reported as infrequent by staff and family; indications of well-being were more frequently observed. Median QOL was the same for staff and family observers (4=“Life is so-so” [7-point item]; 3=“Fair” [5-point item]). Inter-observer assessments of resident QOL (staff vs. family) were correlated (7-point item: r=0.47, ICC=.643; 5-point item: r=0.48, ICC=.645, p<.05 for all). Seven of 18 EOLD-CAD symptoms were significantly positively correlated. ICC values varied between high or moderately high: shortness-of-breath (ICC=.74), choking (ICC=.65), gurgling (ICC=.81), agitation (ICC=.51), fear (ICC=.46), crying (ICC=.65), peace (ICC=.57), and care resistance (ICC=.68) (p<.05 for all). Choking and gurgling were the most prominently reported symptoms by both groups.Early findings provide a contemporary assessment of QOL and symptoms among NH residents with dementia. Measurement properties affirm general reliability and validity of study instruments.

